# Construction of a lipid metabolism-related risk model for hepatocellular carcinoma by single cell and machine learning analysis

**DOI:** 10.3389/fimmu.2023.1036562

**Published:** 2023-03-01

**Authors:** Lisha Mou, Zuhui Pu, Yongxiang Luo, Ryan Quan, Yunhu So, Hui Jiang

**Affiliations:** ^1^ Imaging Department, Shenzhen Institute of Translational Medicine, The First Affiliated Hospital of Shenzhen University, Shenzhen Second People’s Hospital, Shenzhen, China; ^2^ MetaLife Center, Shenzhen Institute of Translational Medicine, The First Affiliated Hospital of Shenzhen University, Shenzhen Second People’s Hospital, Shenzhen, China; ^3^ Department of General Surgery, The First People's Hospital of Qinzhou/The Tenth Affiliated Hospital of Guangxi Medical University, Qinzhou, Guangxi, China

**Keywords:** hepatocellular carcinoma, scRNA-seq, lipid metabolism, prediction model, immune microenvironment, TMB, GSEA, Machine learning

## Abstract

One of the most common cancers is hepatocellular carcinoma (HCC). Numerous studies have shown the relationship between abnormal lipid metabolism-related genes (LMRGs) and malignancies. In most studies, the single LMRG was studied and has limited clinical application value. This study aims to develop a novel LMRG prognostic model for HCC patients and to study its utility for predictive, preventive, and personalized medicine. We used the single-cell RNA sequencing (scRNA-seq) dataset and TCGA dataset of HCC samples and discovered differentially expressed LMRGs between primary and metastatic HCC patients. By using the least absolute selection and shrinkage operator (LASSO) regression machine learning algorithm, we constructed a risk prognosis model with six LMRGs (*AKR1C1*, *CYP27A1*, *CYP2C9*, *GLB1*, *HMGCS2*, and *PLPP1*). The risk prognosis model was further validated in an external cohort of ICGC. We also constructed a nomogram that could accurately predict overall survival in HCC patients based on cancer status and LMRGs. Further investigation of the association between the LMRG model and somatic tumor mutational burden (TMB), tumor immune infiltration, and biological function was performed. We found that the most frequent somatic mutations in the LMRG high-risk group were *CTNNB1*, *TTN*, *TP53*, *ALB*, *MUC16*, and *PCLO*. Moreover, naïve CD8+ T cells, common myeloid progenitors, endothelial cells, granulocyte-monocyte progenitors, hematopoietic stem cells, M2 macrophages, and plasmacytoid dendritic cells were significantly correlated with the LMRG high-risk group. Finally, gene set enrichment analysis showed that RNA degradation, spliceosome, and lysosome pathways were associated with the LMRG high-risk group. For the first time, we used scRNA-seq and bulk RNA-seq to construct an LMRG-related risk score model, which may provide insights into more effective treatment strategies for predictive, preventive, and personalized medicine of HCC patients.

## 1 Introduction

According to recent epidemiological data, 906,000 new cases of liver cancer were diagnosed globally in 2020, making it the sixth most prevalent cancer worldwide (1). Hepatocellular carcinoma (HCC) is a common type of liver cancer (2). The disease is caused by a number of risk factors, including HBV/HCV infection, nonalcoholic steatohepatitis (NASH), alcoholism, and smoking. HCC treatment, including surgery, chemotherapy, and radiation therapy, has significantly enhanced survival and reduced cancer cell proliferation in patients with the disease (3). Early HCC is treatable with tumor resection and liver transplantation; however, many patients are not diagnosed until the late stages (4). As HCC is highly heterogeneous, predictive, preventive, and personalized medicine can improve therapy outcomes. Therefore, it is essential to uncover the mechanisms that drive the progression of HCC, and effective biomarkers must be identified as soon as possible to provide individualized treatment for HCC patients.

Recent studies have revealed that alterations in lipid metabolism are significant metabolic indicators of cancer cells in general (5). Changes in lipid metabolism, for instance, can occur in tumor cells and the tumor microenvironment, which influences the development, proliferation, invasion, and metastasis of cancer cells (6). A previous study showed that TAR DNA-Binding Protein 43 (TDP-43) can suppress apoptosis by facilitating lipid metabolism in HCC (7). In HCC, ovarian cancer, lung adenocarcinoma, pancreatic cancer, renal cell carcinoma, and diffuse glioma, lipid metabolism-related genes (LMRGs) show excellent predictive values (8). Consequently, targeting lipid metabolism has been viewed as a potential way of treating tumors. To date, several prognostic models have examined the value of genes associated with ferroptosis, epithelial-mesenchymal transition, and immunity in HCC, whereas little is known about how LMRGs contribute to HCC and whether LMRGs are correlated with HCC patient prognosis (9–11).

Multiple gene signatures for predicting the prognosis of HCC patients have been created in prior research based on bulk RNA sequencing; however, these signatures have not been used in clinical settings. RNA signals from several cells within a sample are combined during bulk RNA sequencing to reflect the sample’s average RNA content. As a result, cell type predominance has a large impact. However, there are certain genetic traits linked to HCC that may differently favor its development. Therefore, uncommon or diverse cell populations cannot be studied by bulk RNA sequencing. Single-cell RNA sequencing (scRNA-seq), in contrast to bulk RNA sequencing analysis, enables the investigation of transcriptional activity inside a single cell and allows the detection of tiny, clinically important tumor subpopulations (12).

Machine learning (ML) research has increased quickly because it provides a practical means to analyze huge and complicated datasets. In practice, a variety of ML algorithms are used (including random survival forest, support vector machine, gradient boosting, Bayesian, and deep learning). Moreover, these machine learning algorithms have been applied to the clinical management and prevention of HCC (13), including the discovery of biomarkers for early diagnosis (14), the development of prediction signatures for HCC recurrence (15, 16), and the production of single-cell atlases of HCC cell heterogeneity in response to immunotherapy (17).

In this study, we aimed to identify a prognostic biomarker that predicts the overall survival of HCC patients by scRNA-seq and bulk RNA-seq. We identified an LMRG signature in a training HCC cohort and further validated it in an external cohort. A novel nomogram incorporating clinical features and an LMRG signature was also constructed. The results demonstrated that this LMRG signature could help in the early diagnosis of patients with HCC, which also plays essential roles in the prognostication process and could be a viable therapeutic target for HCC patients.

## 2 Methods

### 2.1 Data collection and preparation

TCGA and ICGC provide data on gene expression, prognosis, and clinicopathology for hepatocellular carcinoma (HCC) (18). Single-cell RNA sequencing (scRNA-seq) data from ten HCC patients were downloaded from the Gene Expression Omnibus (19). In addition, a total of 260 HCC samples from the ICGC cohorts (20) with clinical data and 232 with gene expression data were used as independent validation sets. Moreover, lipid metabolism-related genes (LMRGs) were obtained from Reactome.

### 2.2 ScRNA-seq data processing

The transcript count matrix were analyzed with the Seurat package v4.1.0 in R, as mentioned previously. Subsequent analysis was performed for the three specific tumor sites at the primary tumor, portal vein tumor thrombus, and metastatic lymph node in the 10 HCC patients. The resulting matrix was used to select the top 2000 highly expressed and variable genes. These selected genes were then used to compute the independent component (IC). RunUMAP were used to perform expression profile analysis. An absolute value of (|log FC|) > 0.5 and an adjusted *P* value (adj *P*) < 0.05 were used as the cutoff values for differentially expressed genes (DEGs).

### 2.3 Machine learning model construction

We created models utilizing ML algorithms to forecast the prognosis of HCC patients using the TCGA-LIHC dataset. The ML approach, the least absolute shrinkage and selection operator (LASSO) regression model, was chosen based on the distribution of the outcome variable. The LASSO regression approach automates the selection of variables by shrinkage and the deletion of nonsignificant variables by setting them to zero to achieve L1 regularization to maximize prediction accuracy (21). By minimizing the sum of squares, LASSO adjusts a shrinkage penalty lambda (λ) or tuning hyperparameter to the regression coefficients. As lambda values increase, the model becomes more biased, and further coefficients may be eliminated or set to zero. Using the optimum lambda value, the parameter estimates for the prediction model were calculated. We fitted additional models using a negative binomial depending on the number of zeros in the outcome variable. To determine which factors had the highest predictive value for determining the prognosis of HCC, we assessed the variable relevance rankings. The variables with the highest predictive value were identified among those chosen by the model with the best lambda value. The LMRG signature was then determined using the risk score (RS) formula based on the findings of the multivariate COX regression: RS = ∑ (βi * Expi). The accuracy of the risk score model was evaluated by ROC and Kaplan–Meier survival analyses. The Kaplan–Meier survival curve combined with the log-rank test was employed to evaluate the survival differences between the LMRG high- and low-risk groups. The model was further validated in an external dataset of ICGC.

### 2.4 Nomogram construction

Univariate and multivariate regression analyses were used to analyze the independent clinical factors. Prior to nomogram construction, the LMRG signature and the clinical characteristics were integrated. The predictive accuracy of the prognostic model was evaluated by the time-dependent ROC curve. We evaluated the performance of the established nomogram on the basis of ROC curves, and decision curve analysis for overall survival at 1, 3, and 5 years.

### 2.5 Histological data analysis of HCC

The protein expression of AKR1C1, CYP27A1, CYP2C9, GLB1, HMGCS2, and PLPP1 in HCC patients was analyzed using histological data from the Human Protein Atlas (HPA) database (http://www.proteinatl.as.org/).

### 2.6 Western blot

HCC samples from all patients were collected with written informed consent with approval from the institutional research ethics committee of the First People’s Hospital of Qinzhou (Approval number, 2021-15). The patients provided their written informed consent to participate in this study. Total proteins were extracted by lysis buffer radioimmunoprecipitation assay (RIPA; Beyotime, USA) with 1% phenylmethylsulfonyl fluoride (PMSF). Polyvinylidene fluoride (PVDF) membranes containing proteins separated on sodium dodecylsulfate–polyacrylamide gel electrophoresis (SDS-PAGE) gels were blocked with 5% nonfat milk and incubated with different primary antibodies, including β-ACTIN (#4970S, Cell Signal Technology), CYP27A1 (#67045-1-Ig, Proteintech), and GLB1 (#TA505544, ORIGENE), overnight at 4°C, and chemiluminescence was used for detection. Protein quantification was performed using ImageJ software.

### 2.7 Tumor mutational burden

The DNA somatic mutation dataset from TCGA-LIHC was used to determine whether LMRG signatures were associated with TMB. In accordance with our previous description, HCC patients were divided into LMRG-high and LMRG-low risk groups (https://www.gsea-msigdb.org/gsea/msigdb/index.jsp). The somatic mutations in the two LMRG groups were further visualized using the “maftools” R package.

### 2.8 Gene set enrichment analysis

To further understand the relationship of LMRGs and biological processes, we used GSEA software based on the Kyoto Encyclopedia of Genes and Genomes (KEGG) gene set (KEGG C2, MSigDB database v7.5.1) to assess possible differences in biological functions between the LMRG high- and low-risk groups (https://www.gsea-msigdb.org/gsea/msigdb/index.jsp). To measure the significance of genomic enrichment, we used the *P* value of normalized enrichment scores and the *Q*-value of FDR.

### 2.9 Immune infiltration

To analyze the association between LMRG signatures and tumor-infiltrating immune cells, xCell analysis was used to estimate the fraction of the 64 subtype immune cells in each TCGA-LIHC sample (22). Subgroup analysis of signature immune cells for both LMRG high- and low-risk patients was carried out. An illustration of the results is shown using a heatmap and violin plot.

### 2.10 Statistical analysis

All analyses were performed with R version 4.0.5 and its appropriate packages without special instructions. A *P* value < 0.05 was set as statistically significant for all the analyses.

## 3 Results

### 3.1 Data profiling of the GSE149614 cohort by scRNA-seq

**Figure 1:**
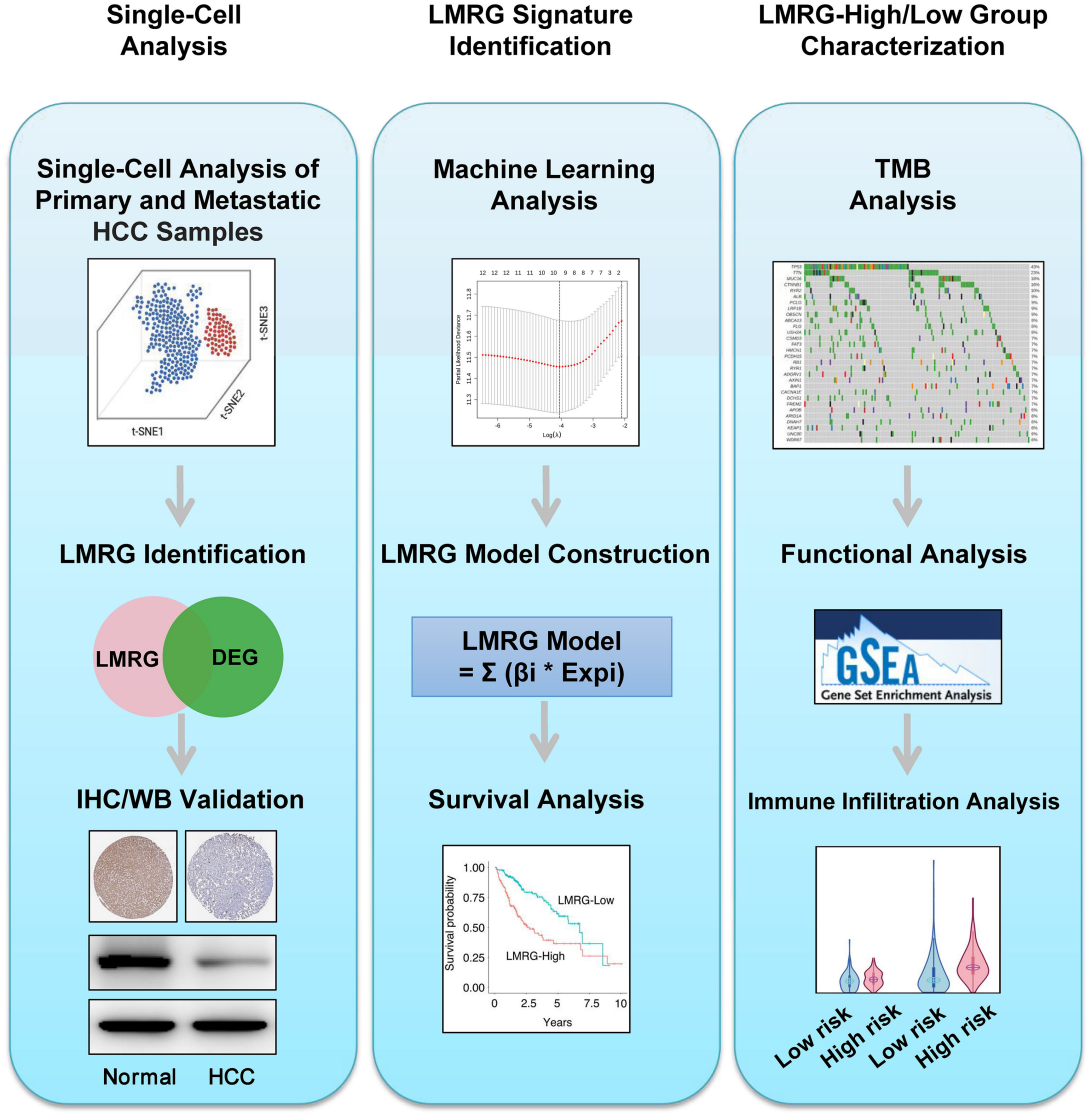
Workflow of this study. Step 1: Single-cell analysis. Step 2: LMRG signature identification. Step 3: LMRG-high and -low risk group characterization.

**Figure 2:**
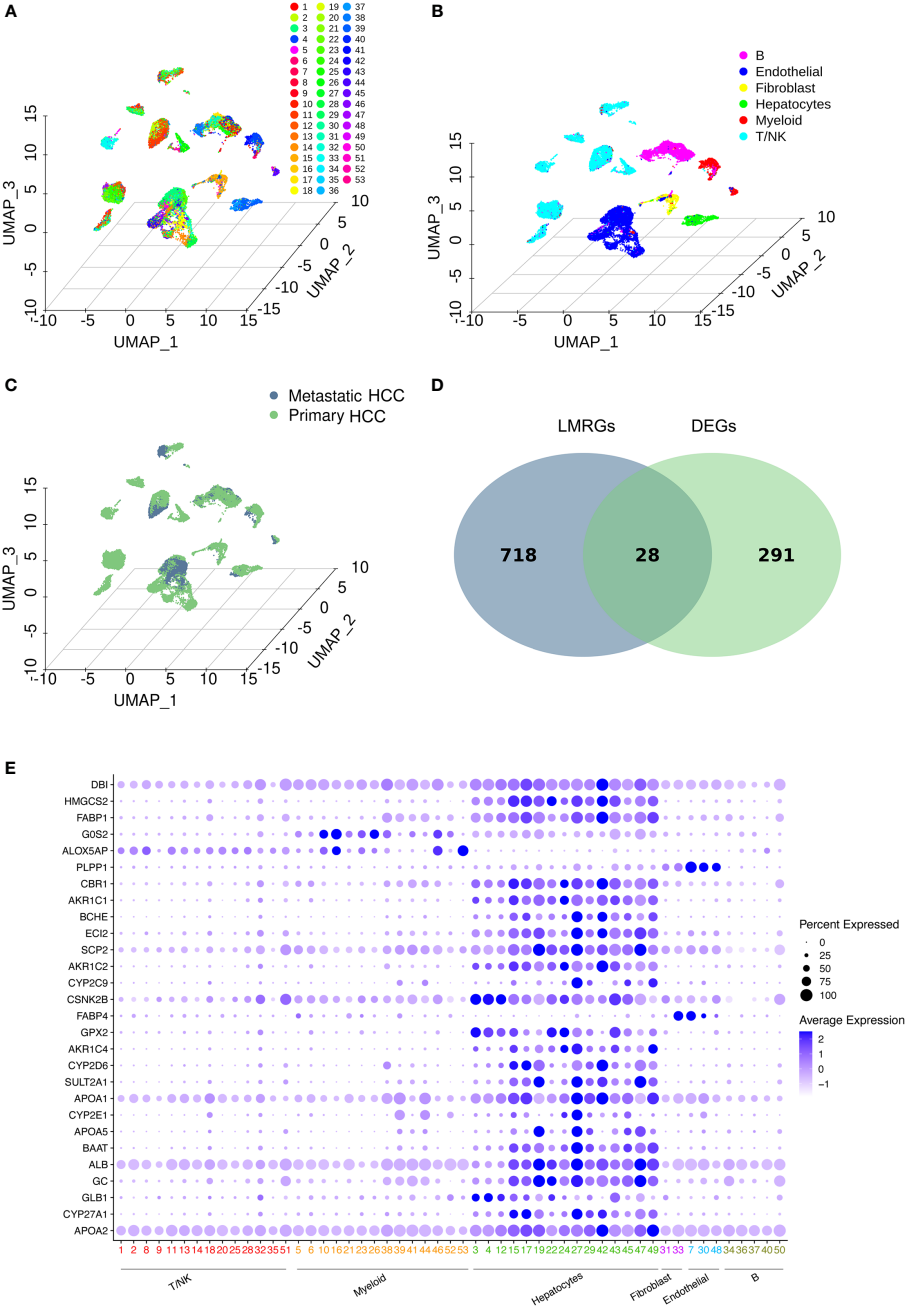
Data profiling of the GSE149614 cohort by scRNA-seq. **(A-C)** Samples from primary and metastatic hepatocellular carcinoma (HCC) tissue clustered by the UMAP algorithm. The dimension reduction showed the results of **(A)** 53 clusters, **(B)** six major cell types, and **(C)** primary and metastatic HCC. **(D)** Identification of lipid metabolism-related genes (LMRGs) in HCC development. A total of 746 LMRGs were assessed in the Reactome Pathway Database (https://reactome.org/). A total of 319 differentially expressed genes (DEGs) between primary and metastatic HCC samples were used to intersect with the 746 LMRGs. Finally, 28 screened LMRGs were identified. **(E)** Dot plot showing the expression of 28 LMRGs in different cell types.

The workflow of this study is shown in **Figure 1**. After downloading the GSE149614 cohort from the GEO database, the data were profiled using scRNA-seq to determine the differentially expressed genes (DEGs) between primary and metastatic tumor tissues in HCC (19). UMAP algorithms were implemented for nonlinear dimensionality reduction, and samples were clustered, as shown in **Figures 2A–C**. The samples included 53 clusters (**Figure 2A**) and six major cell types (**Figure 2B**). Among the six major cell types, hepatocytes are the most abundant cells. Myeloid cells, T/NK cells, B cells, fibroblast cells, and endothelial cells are less abundant cells. We also showed the distribution of cells in primary HCC and metastatic HCC (**Figure 2C**).

Then, DEGs related to HCC development were identified. To identify lipid metabolism-related genes (LMRGs) in HCC development, the DEGs were intersected with the LMRGs in the Reactome Pathway Database (https://reactome.org/), and 28 genes were obtained (**Figure 2D** and **Supplementary Table 1**). The 28 genes were defined as the screened LMRGs. **Figure 2E** shows the expression of 28 genes in different cell types. Most of the 28 genes were highly expressed in hepatocytes. However, *G0S2* and *ALOX5AP* were highly expressed in myeloid cells, *PLPP1* was highly expressed in endothelial cells, and *FABP4* was highly expressed in fibroblasts and endothelial cells.

### 3.2 Identification and validation of prognostic biomarkers for the risk model

**Figure 3:**
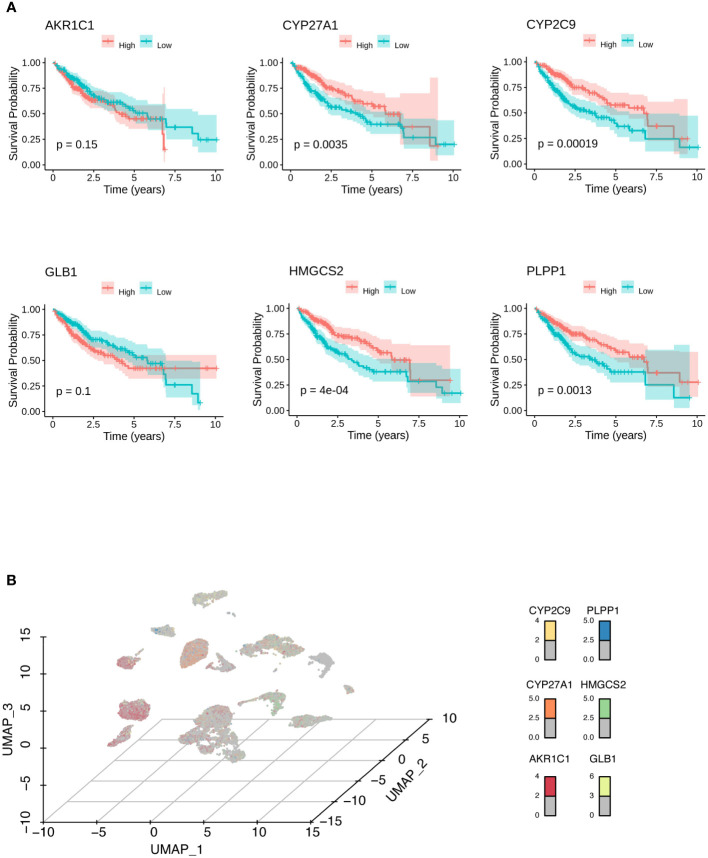
Identification of biomarkers for HCC. **(A)** Kaplan-Meier analysis showed the survival probabilities of HCC patients with high and low expression of the hub LMRG gene (*AKR1C1*, *CYP27A1*, *CYP2C9*, *GLB1*, *HMGCS2*, and *PLPP1*). **(B)** UMAP results showed the expression of the hub LMRG genes (*AKR1C1*, *CYP27A1*, *CYP2C9*, *GLB1*, *HMGCS2*, and *PLPP1*) in single-cell data.

**Figure 4:**
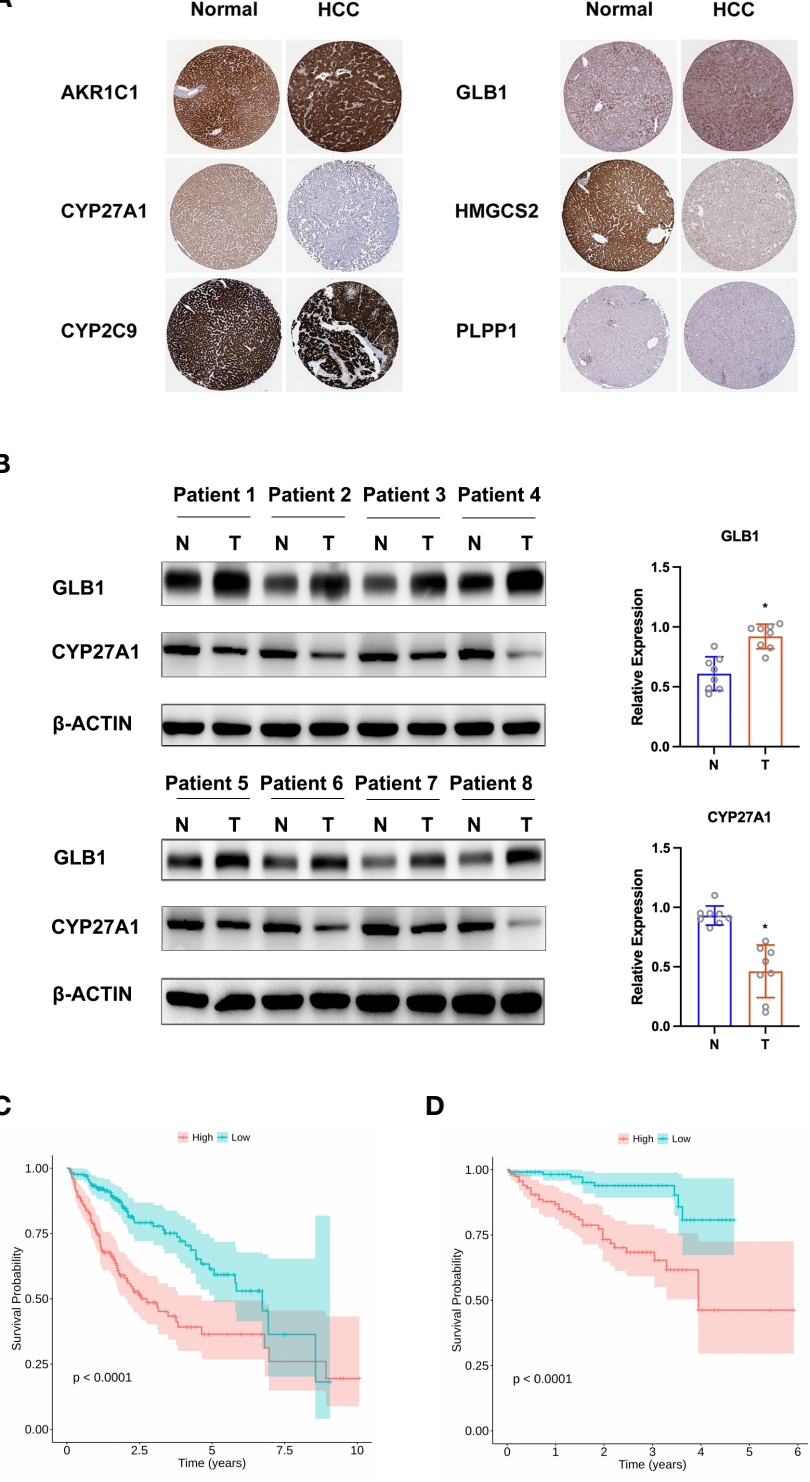
Validation of hub LMRG genes. **(A)** Histological data of AKR1C1, CYP27A1, CYP2C9, GLB1, HMGCS2, and PLPP1 from the Human Protein Atlas. **(B)** Western blotting analysis of GLB1 and CYP27A1. **(C)** Kaplan-Meier curves of the risk model in the training cohort. **(D)** Kaplan-Meier curves of the risk model in the testing cohort. Training cohort: TCGA-LIHC; testing cohort: ICGC-LIRI-JP. *p<0.05.

To construct a predictive risk model, 28 previously screened LMRGs were subjected to univariate Cox regression analysis and the LASSO method. **Supplementary Table 2** details the univariate Cox analysis of the 28 LMRGs. The LASSO model in **Supplementary Figures 1A, B** implies that eight genes (*AKR1C1*, *APOA1*, *CYP27A1*, *CYP2C9*, *GC*, *GLB1*, *HMGCS2*, and *PLPP1*) would be assessed in multivariate Cox analysis. As a result, *AKR1C1*, *CYP27A1*, *CYP2C9*, *GLB1*, *HMGCS2*, and *PLPP1* were selected for the LMRG signature (**Supplementary Table 2**), and the risk score was risk score (RS) = 0.00225 × *AKR1C1* - 0.00200 × *CYP27A1* - 0.00182 × *CYP2C9* + 0.02063 × *GLB1* - 0.00074 × *HMGCS2* - 0.01185 × *PLPP1*. Based on Kaplan-Meier analysis, we calculated survival probabilities for HCC patients with high and low expression of each gene (*AKR1C1*, *CYP27A1*, *CYP2C9*, *GLB1*, *HMGCS2*, and *PLPP1*). The results showed that high expression of *CYP27A1, CYP2C9, HMGCS2*, and *PLPP1* was correlated with better overall survival (OS) outcomes (**Figure 3A**). The expression of these genes in single-cell data (GSE149614) is shown in **Figures 3B**. Moreover, to examine the protein levels of AKR1C1, CYP27A1, CYP2C9, GLB1, HMGCS2, and PLPP1 between HCC and normal samples, we used the histological data in the HPA database and identified that the protein expression of AKR1C1 and GLB1 was significantly higher in HCC than in normal patients, while the protein expression of CYP27A1 and HMGCS2 was significantly higher in normal samples than in HCC samples (**Figure 4A**). Previous studies showed that AKR1C1 was upregulated in HCC and HMGCS2 was downregulated in HCC (23–25), which was consistent with our results. Since AKR1C1 and HMGCS2 were already reported in previous studies, we further validated the expression of GLB1 and CYP27A1 by Western blotting in HCC samples and adjacent normal samples. The results were consistent with the IHC results, as shown in **Figure 4B**. Subjects in the training cohort (TCGA-LIHC) and the testing cohort (ICGC-LIRI-JP) were divided into LMRG high- and low-risk groups by the median RS (**Supplementary Table 3**). The median RS for TCGA-LIHC was 1.001, and the median RS for ICGC-LIRI-JP was 0.976. Moreover, the areas under the ROC curves (AUCs) were evaluated, resulting in finding the AUCs of OS. The values for the training cohort were 0.745 (1 year), 0.696 (3 years), and 0.702 (5 years), while the values for the testing cohort were 0.792 (1 year), 0.79 (3 years), and 0.761 (5 years) (**Supplementary Figures 2A, B**). The survival probabilities of HCC patients in both the training and testing cohorts were estimated by Kaplan-Meier analysis. **Figures 4C, D** show that poorer overall survival outcomes were observed in the LMRG high-risk groups than in the LMRG low-risk groups.

### 3.3 Construction and validation of the nomogram

**Figure 5:**
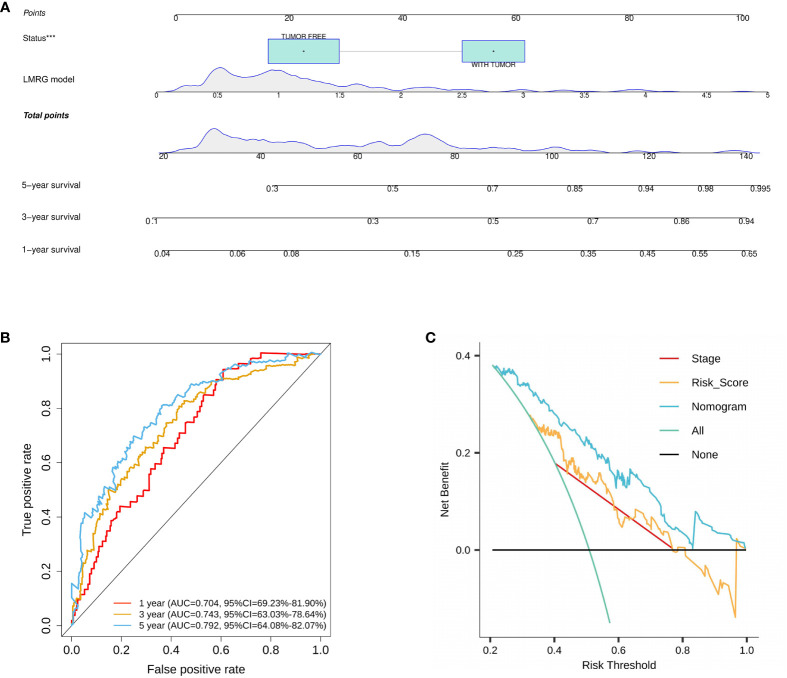
Construction and validation of a nomogram for HCC. **(A)** A nomogram was constructed based on cancer status and six LMRGs. **(B)** ROC curves of the nomogram in the training cohort. **(C)** Decision curve analysis of the nomogram in the TCGA cohort at 5 years. ***p<0.001.

On the basis of the training cohort, univariate and multivariate Cox regression models were utilized to screen significantly correlated clinical parameters for prognosis (**Supplementary Figures 3A, B**). As a result, cancer status and RS were screened (**Supplementary Figures 3A, B**). Then, a nomogram involving cancer status and RS was developed, and the survival of HCC patients at 1, 3, and 5 years was predicted by summarizing all points of the clinical parameters (**Figure 5A**). High AUC values (0.704, 0.743, and 0.792 for 1-, 3-, and 5-year survival, respectively) implied that the nomogram performed well in predicting OS (**Figure 5B**). To estimate the prediction power of the nomogram constructed, a decision curve analysis was performed. The results revealed that the nomogram could provide better benefits to HCC patients than the risk model constructed by genes for 5-year OS prediction (**Figure 5C**).

### 3.4 Mutation analysis in the training cohort

**Figure 6:**
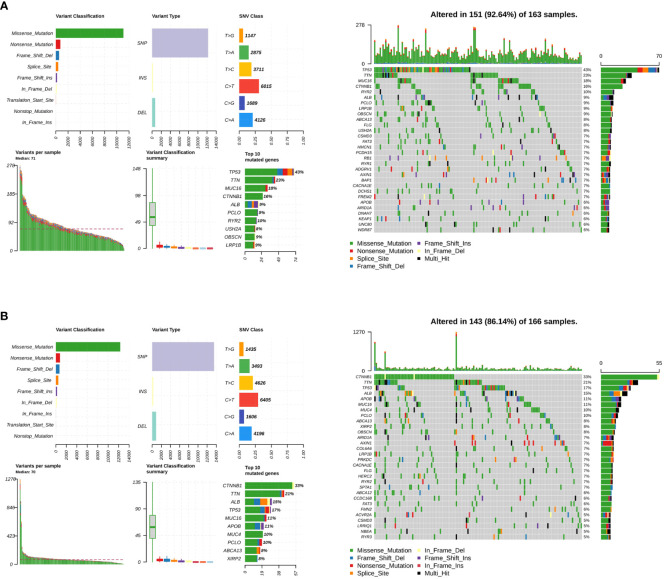
The tumor mutation burden characteristics of patients in the LMRG high- and low-risk groups. **(A)** Mutational landscape in the TCGA cohort of the LMRG high-risk groups. **(B)** Mutational landscape in the TCGA cohort of the low-risk groups.

Somatic mutations were evaluated to analyze the tumor mutation burden and RS. The mutational landscape was constructed, indicating that the LMRG high-risk group (92.64%) had more frequent mutation events than the low-risk group (86.14%) (**Figure 6A**). In addition, three significantly mutated genes, *TP53* (LMRG high-risk: 43%, low-risk: 17%), *CTNNB1* (LMRG high-risk: 16%, low-risk: 33%), and *TTN* (LMRG high-risk: 23%, low-risk: 21%), were identified in the two groups (**Figure 6B**. Accordingly, it was revealed that a mutation event was a risk factor in HCC.

### 3.5 Gene set enrichment analysis in the training cohort

**Figure 7:**
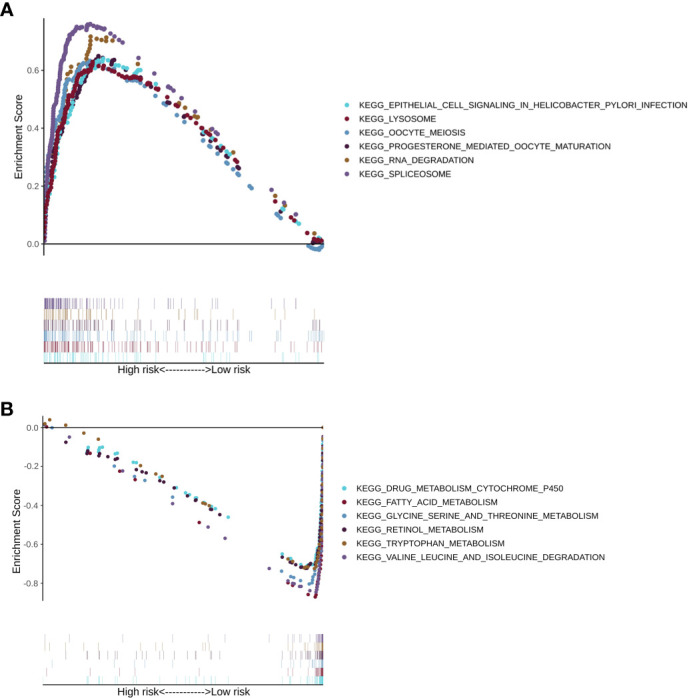
The KEGG pathway enrichment analysis of patients in the LMRG high- and low-risk groups. **(A)** Enriched pathways in the LMRG high-risk group. **(B)** Enriched pathways in the LMRG low-risk group.

Next, gene set enrichment analysis provided the pathways that were enriched in the LMRG high- and low-risk groups of the training cohorts. Pathways such as RNA degradation, spliceosome, epithelial cell signaling in Helicobacter pylori infection, lysosome, oocyte meiosis, and progesterone-mediated oocyte maturation were upregulated in the LMRG high-risk group (**Figure 7A**). On the other hand, pathways such as fatty acid metabolism, drug metabolism-cytochrome P450, glycine, serine and threonine metabolism, retinol metabolism, valine, leucine and isoleucine degradation, and tryptophan metabolism were upregulated in the LMRG low-risk group (**Figure 7B**). Moreover, other enriched pathways are illustrated in **Supplementary Table 4**.

### 3.6 Analysis of immune microenvironment characteristics in the training cohort

**Figure 8:**
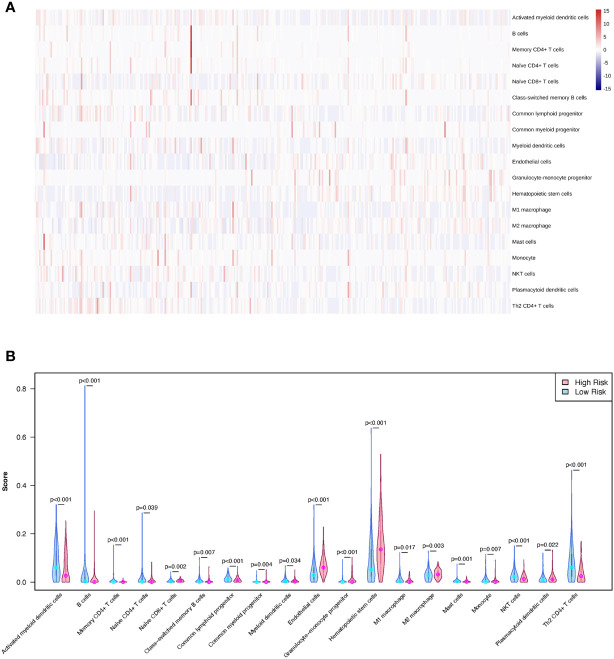
The different immune infiltration of patients in the LMRG high- and low-risk groups were identified. **(A)** The profile of immune infiltration in the TCGA-LIHC cohort showed by heatmap. **(B)** The violin plot shows the significantly different immune cells between the two risk groups in the TCGA cohort.

A training cohort was implemented in the xCell algorithm for immune infiltration estimation (**Supplementary Table 5**). The results of the immune infiltration in the TCGA-LIHC cohort were shown in **Figure 8A**. Remarkably, high levels of naïve CD8+ T cells, common myeloid progenitors, endothelial cells, granulocyte-monocyte progenitors, hematopoietic stem cells, M2 macrophages, and plasmacytoid dendritic cells were observed in the LMRG high-risk group. However, high levels of activated myeloid dendritic cells, B cells, memory CD4+ T cells, class-switched memory B cells, common lymphoid progenitors, myeloid dendritic cells, M1 macrophages, mast cells, monocytes, NKT cells, and Th2 CD4+ T cells were observed in the LMRG low-risk group (**Figure 8B**).

## 4 Discussion

Hepatocellular carcinoma (HCC), one of the most common and aggressive tumors, has been linked to a high rate of morbidity for patients. Although several environmental or genetic risk factors linked to hepatocellular carcinoma (HCC) have been identified, the molecular processes causing HCC occurrence are still unknown. The proliferation and spread of tumors are facilitated by abnormal lipid metabolism. Research on the mechanism of lipid metabolism might therefore aid in the development of novel, targeted treatments to control or remove these refractory tumor cells, which might lead to the development of new medicines for HCC. A great number of studies have found a link between abnormal lipid metabolism and the onset and progression of malignancies (26). As a result, a lipid metabolism-related gene (LMRG) signature for predicting the survival of HCC patients is needed. Large-scale bulk and single-cell sequencing of tumor samples is now possible because of recent breakthroughs in sequencing technology. Moreover, the direct examination of genetic cell-to-cell variety is made possible by machine learning technology. For the first time, we were able to create a 6-LMRG signature of HCC in this study by scRNA-Seq. *AKR1C1, CYP27A1, CYP2C9, GLB1, HMGCS2*, and *PLPP1*, all six LMRGs, have been implicated in the genesis and progression of cancer. Upregulated *AKR1C1* expression was found in *HPV16*-positive oropharyngeal squamous cell carcinoma with viral integration, and it was linked with a poor prognosis in both HPV-positive and HPV-negative tumors (27). Furthermore, as one of the vitamin D pathway genes, *CYP27A1* has some impact on prostate cancer chemoprevention based on vitamin D metabolism and has the ability to predict the prognosis of prostate cancer patients (28, 29). A variation in the *CYP2C9* gene has been linked to an increased risk of colorectal cancer and adenoma (30). *GLB1* is a lysosomal exoglycosidase that catabolizes glycoconjugates and has been linked to cancer cell senescence (31). *HMGCS2* has been linked to oncogenic activity in a variety of human tumors (32, 33). *HMGCS2* was identified as a differential hub gene of lipid metabolism in the pancancer immune microenvironment. Lower levels of *PLPP1* mRNA expression in tumor tissues than in surrounding normal tissues are linked to a worse prognosis (34).

Using a multivariate Cox regression analysis approach, we combined the signatures of several genes. The nomogram model, composed of the tumor status and the risk score derived from the LMRG signature, can visually predict the one-, three- and five-year overall survival outcomes for individual HCC patients. The final six genes demonstrated high accuracy in both the validation set and the overall prognosis for samples. In both TCGA and ICGC data, the LMRG low-risk score group showed worse results than the LMRG high-risk score group. In our present study, the most frequent somatic mutations in the LMRG high-risk group were *CTNNB1, TTN, TP53, ALB, MUC16*, and *PCLO.* Previous studies have shown that *TP53*, *MUC16*, and *TTN* mutations are common in many types of cancer, including gastric cancer and pancreatic and bladder cancers, and are associated with poor prognoses (35–38). In our study, the immune microenvironments of the LMRG high- and low-risk groups were analyzed by the xCell algorithm. Here, naïve CD8^+^ T cells, common myeloid progenitors, endothelial cells, granulocyte-monocyte progenitors, hematopoietic stem cells, M2 macrophages, and plasmacytoid dendritic cells were significantly correlated with the LMRG high-risk group, which was first revealed in an HCC study.

The results of the gene set enrichment analysis showed that fatty acid metabolism and lysosome pathways, which involve lipid metabolism-related genes, changed between the LMRG high-risk group and the LMRG low-risk group. The fatty acid metabolism pathway participates in energy production, membrane synthesis, and signal transduction in tumor initiation and progression. Cancer cells rely on fatty acids as cellular building blocks for membrane formation, energy storage, and the production of signaling molecules (39). Lysosome pathways were associated with the LMRG high-risk group in our study. Lysosome pathways play an important role in autophagy. Autophagy, which is an evolutionarily conserved cellular degradation process that delivers cellular components to lysosomes, plays a critical role in cellular homeostasis through the degradation of lipids (40). Dysfunction or dysregulation of autophagy has been proven to be associated with HCC (41). The lysosome is a metabolic signaling hub that integrates different environmental signals to regulate core anabolic and catabolic pathways critical in the maintenance of cellular homeostasis (42). As a result, the changes in fatty acid metabolism and lysosome pathways between the LMRG high-risk group and the LMRG low-risk group affect the overall survival outcomes of HCC patients through cellular homeostasis.

The findings of our study highlight the significant role of multiomics studies in basic research as well as translational and applied research within the field of personalized medicine for HCC. In particular, biomarkers based on LMRGs are essential for a reliable and effective evaluation of HCC prognosis and diagnosis. Moreover, clarifying molecular mechanisms through LMRGs is essential for the discovery of effective targets to treat HCC personalized treatment. HCC personalized medicine will benefit greatly from the prognosis-related LMRGs, and risk models identified in this study.

## Data availability statement

The original contributions presented in the study are included in the article/**Supplementary Material**. Further inquiries can be directed to the corresponding author.

## Ethics statement

The studies involving human participants were reviewed and approved by the institutional research ethics committee of the First People’s Hospital of Qinzhou. The patients/participants provided their written informed consent to participate in this study.

## Author contributions

LM and ZP initiated the study. RQ performed the analysis. RQ and YS wrote and revised the manuscript. YL collected the patient samples. LM, ZP, and HJ designed the study and revised the manuscript. All authors contributed to the article and approved the submitted version.

## Supplementary material

Supplementary Figure 1 LASSO regression analysis of the LMRGs. **(A)** LASSO coefficient profiles of eight prognostic LMRGs. **(B)** Partial likelihood deviance curves of eight prognostic LMRGs.

Supplementary Figure 2 ROC curves of the LMRG model. **(A)** ROC curves of the risk model in the training cohort. **(B)** ROC curves of the risk model in the testing cohort. Training cohort: TCGA-LIHC; testing cohort: ICGC-LIRI-JP.

Supplementary Figure 3 Cox regression analysis of clinical parameters. **(A)** Univariate analysis of clinical parameters. **(B)** Multivariate analysis of clinical parameters.
