# Supplementary figures and images for: Construction of a lipid metabolism-related risk model for hepatocellular carcinoma by single cell and machine learning analysis
Source: Front Immunol. 2023 Mar 1;14:1036562. doi: 10.3389/fimmu.2023.1036562 (PMC10014552; doi:10.3389/fimmu.2023.1036562)

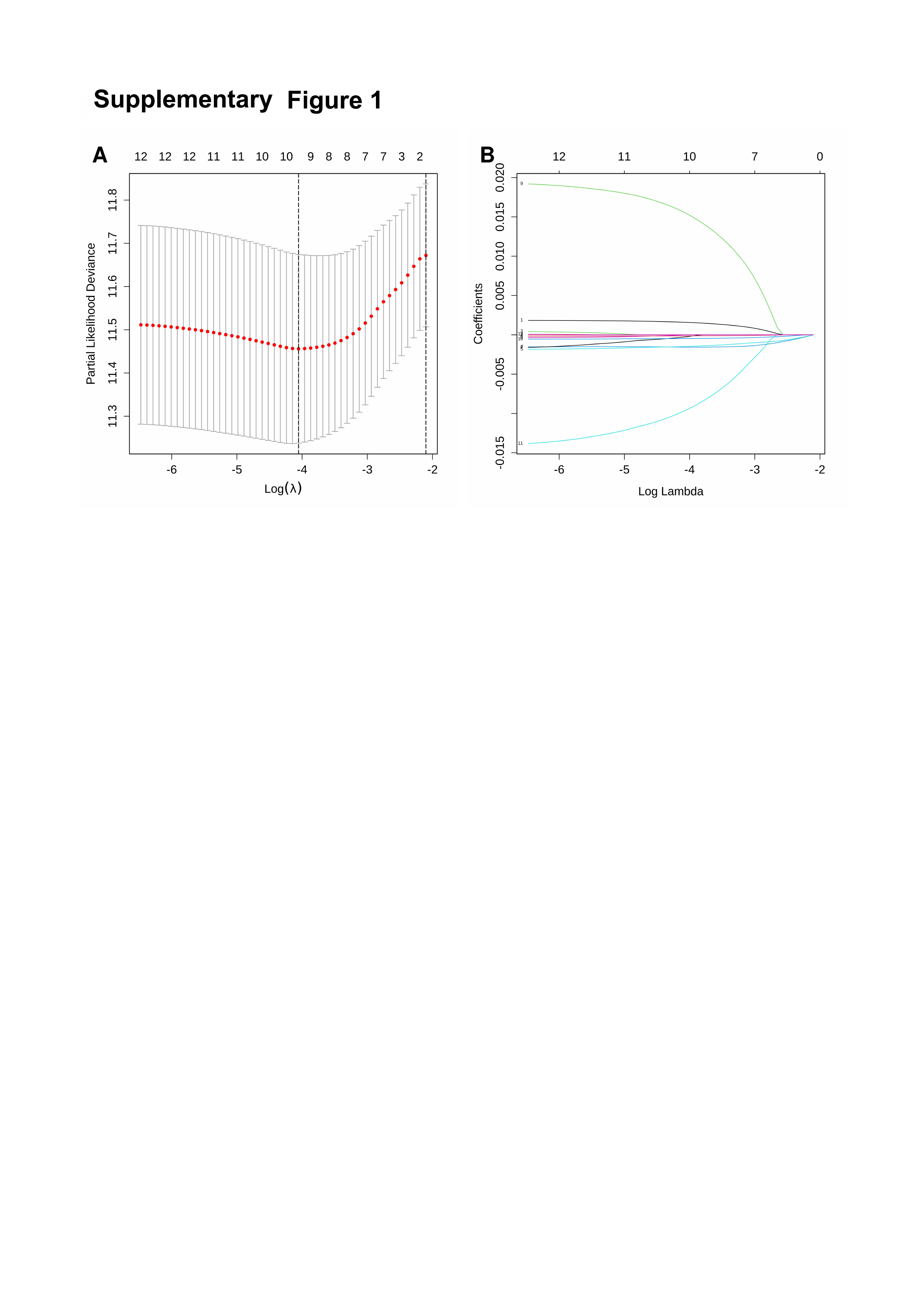

Supplement: Supplementary Figure 1 — LASSO regression analysis of the LMRGs. (A) LASSO coefficient profiles of eight prognostic LMRGs. (B) Partial likelihood deviance curves of eight prognostic LMRGs. [file Image_1.tif]

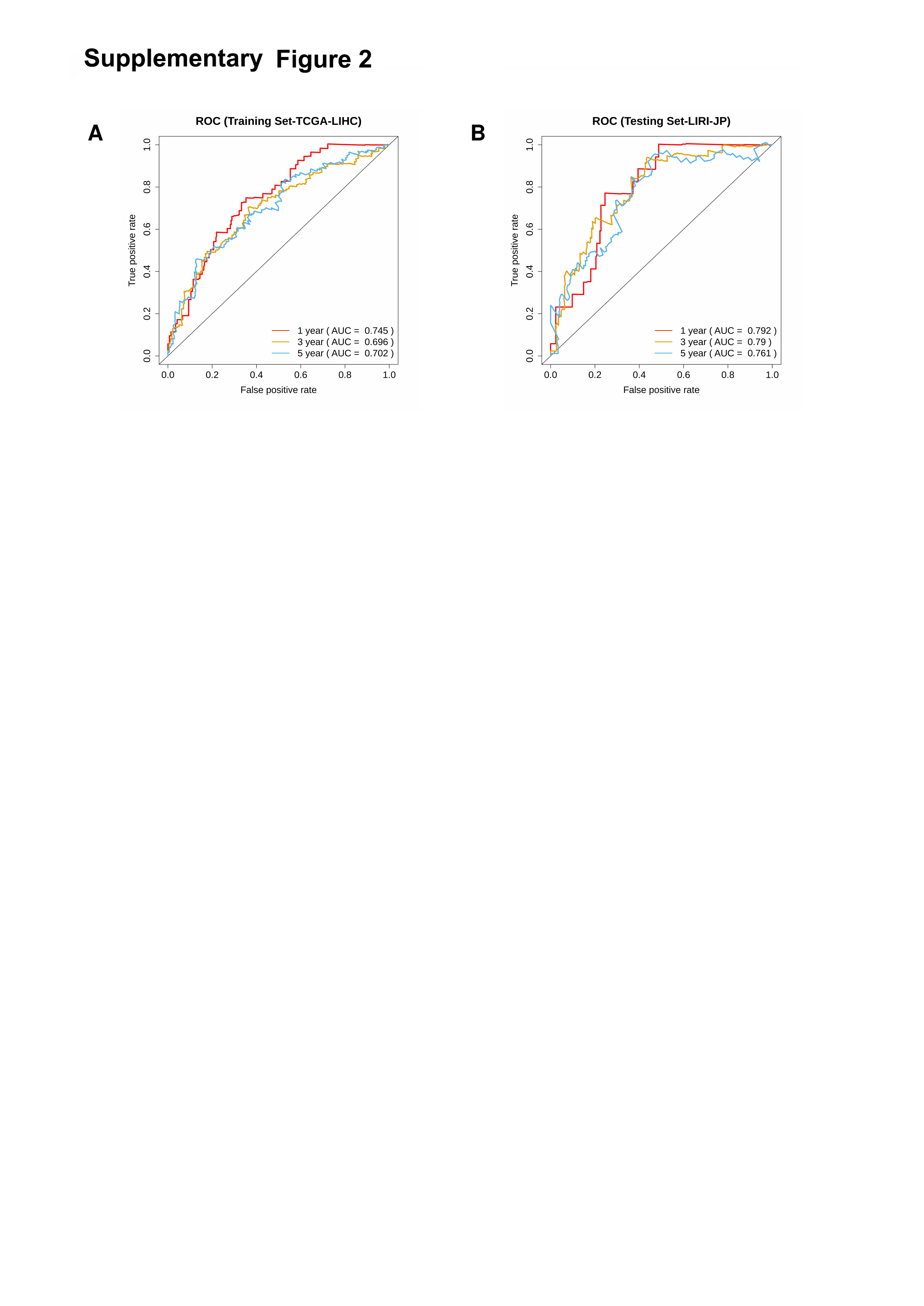

Supplement: Supplementary Figure 2 — ROC curves of the LMRG model. (A) ROC curves of the risk model in the training cohort. (B) ROC curves of the risk model in the testing cohort. Training cohort: TCGA-LIHC; testing cohort: ICGC-LIRI-JP. [file Image_2.tif]

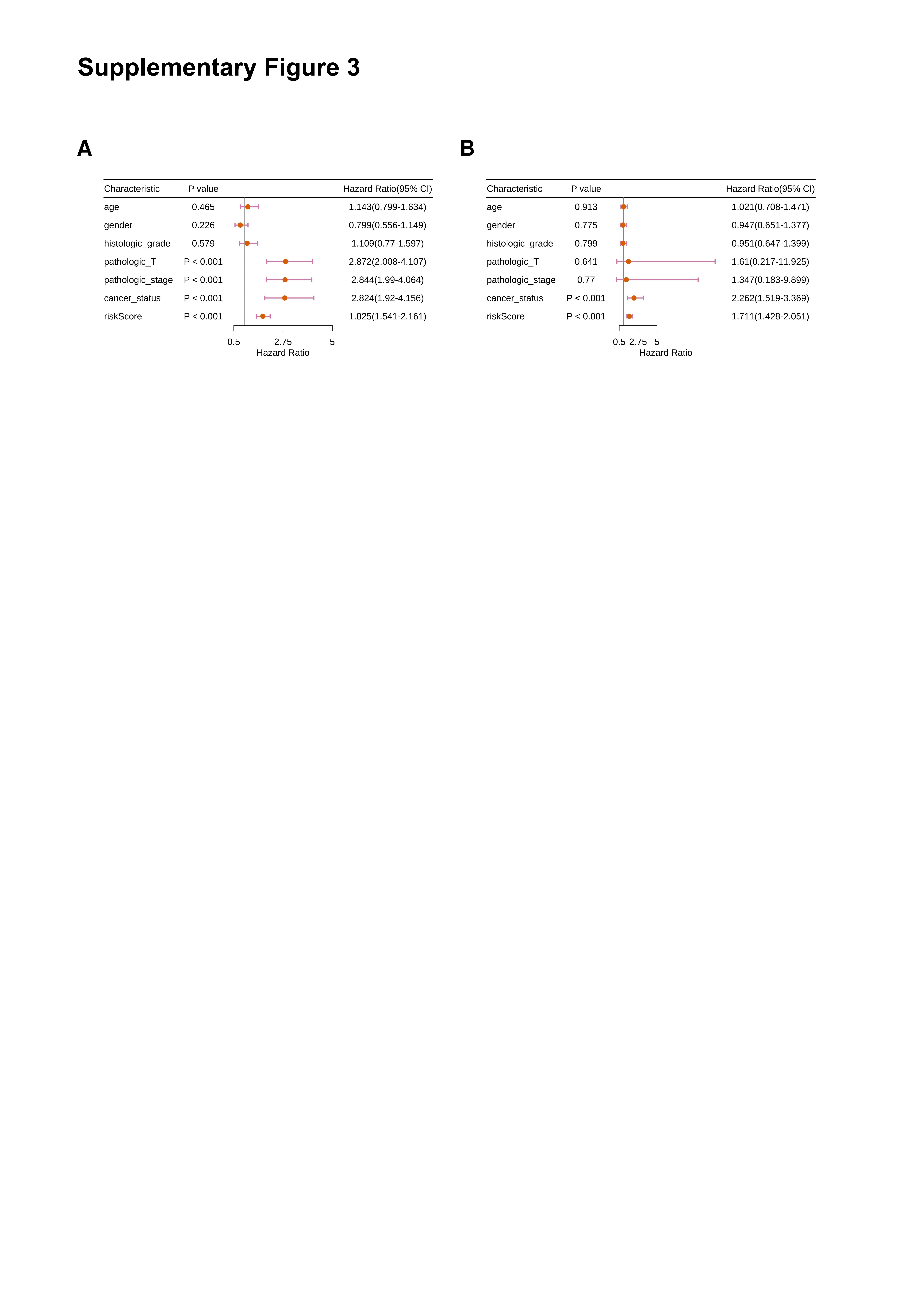

Supplement: Supplementary Figure 3 — Cox regression analysis of clinical parameters. (A) Univariate analysis of clinical parameters. (B) Multivariate analysis of clinical parameters. [file Image_3.tif]
